# *In vitro* and *in vivo* anti-leukemic activity of the peptidase-potentiated alkylator melflufen in acute myeloid leukemia

**DOI:** 10.18632/oncotarget.13856

**Published:** 2016-12-10

**Authors:** Sara Strese, Hassan Bashir Saadia, Ebba Velander, Caroline Haglund, Martin Höglund, Rolf Larsson, Joachim Gullbo

**Affiliations:** ^1^ Department of Medical Sciences, Division of Cancer Pharmacology and Computational Medicine, Uppsala University, Uppsala, Sweden; ^2^ Department of Immunology, Genetics and Pathology, Uppsala University, Uppsala, Sweden; ^3^ Department of Medical Sciences, Division of Hematology, Uppsala University, Uppsala, Sweden

**Keywords:** melflufen, drug development, alkylator, pre-clinical, acute myeloid leukemia

## Abstract

The novel aminopeptidase potentiated alkylating agent melflufen, was evaluated for activity in acute myeloid leukemia in a range of *in vitro* models, as well as in a patient derived xenograft study. All tested AML cell lines were highly sensitive to melflufen while melphalan was considerably less potent. In the HL-60 cell line model, synergy was observed for the combination of melflufen and cytarabine, an interaction that appeared sequence dependent with increased synergy when melflufen was added before cytarabine. Also, in primary cultures of AML cells from patients melflufen was highly active, while normal PBMC cultures appeared less sensitive, indicating a 7-fold *in vitro* therapeutic index. Melphalan, on the other hand, was only 2-fold more potent in the AML patient samples compared with PBMCs. Melflufen was equally active against non-malignant, immature CD34^+^ progenitor cells and a more differentiated CD34^+^ derived cell population (GM14), whereas the stem cell like cells were less sensitive to melphalan. Finally, melflufen treatment showed significant anti-leukemia activity and increased survival in a patient derived xenograft of AML in mice. In conclusion, melflufen demonstrates high and significant preclinical activity in AML and further clinical evaluation seem warranted in this disease.

## INTRODUCTION

Acute myeloid leukemia (AML) is an aggressive blood malignancy originating from hematopoietic blast cells in the bone marrow, with maturation arrest of the myeloid cells resulting in hematopoietic insufficiency such as cytopenia or anemia [1]. According to the Swedish Acute Leukemia Registry, median age for adult AML patients was 72 years in 2009 [2]. The cause of AML is usually unknown, but 20% of all patients have a history of a previous chronic bone marrow diseases, or prior radiation or chemotherapy. AML progresses rapidly and is fatal without specific therapy where complete remission (CR) is required for long-term survival [3]. As for many other malignant diseases combination chemotherapy (for AML often an anthracycline and cytarabine) is needed to maximize tumor-killing effect and reduce drug specific toxicity.

Melphalan flufenamide (melflufen, chemical name L-melphalanyl-p-L-fluorophenylalanine ethyl ester hydrochloride, previously designated J1) is a peptidase-potentiated alkylating agent (Supplementary Figure 1). Under the action of aminopeptidases, like aminopeptidase N [4], melflufen is hydrolyzed, leading to high intracellular concentrations of alkylating moieties (e.g. melphalan), able to interact with nucleic acids within the tumor cells [5]. The ubiquitous enzyme Aminopeptidase N (APN)/CD13 is an ectopeptidase involved in cellular processes that contribute to uncontrolled cell growth, invasion, metastasis and angiogenesis, [6–9]. Increased APN expression has been shown to be associated with a malignant phenotype of several human cancers, including AML [6, 10, 11].. In AML, APN is and is expressed on stem cells and leukemic blasts [6, 10], on the cell surface [11, 12] and in the cytoplasm [12], and recently occurrence of APN was also demonstrated in microvesicles from patients with myeloid tumors [13]. Since APN mediates cleavage of melflufen to its parental drug melphalan in the tumor cell cytoplasm, or in environment with high concentration of APN, a clinical usability of melflufen in AML is suggested [4].

Melflufen is presently being investigated in a successful phase I/IIa clinical trial in multiple myeloma patients [14] and has also received an orphan drug designation in the treatment of relapsed and refractory multiple myeloma by EMA and FDA [15]. Since melflufen has shown to be more active than melphalan in various *in vivo* and *in vitro* models [5, 16–20], without corresponding increase in toxicity, the therapeutic index of melflufen appears to be superior to that of melphalan. Melphalan is currently in clinical use in various standard combination chemotherapy protocols, such as MP (melphalan/prednisone) in multiple myeloma, or BEAM (BCNU/etoposide/Ara-C/melphalan) in lymphomas, also suggesting an important role for melflufen in combination therapy.

Although AML survival has improved, in particular among younger patients, the majority of patients still die from the disease, and there is still need for new effective drugs, single or in combination. Here we will characterize the activity of melflufen in AML *in vitro* and *in vivo*.

## RESULTS

### AML cell lines

**Figure 1:**
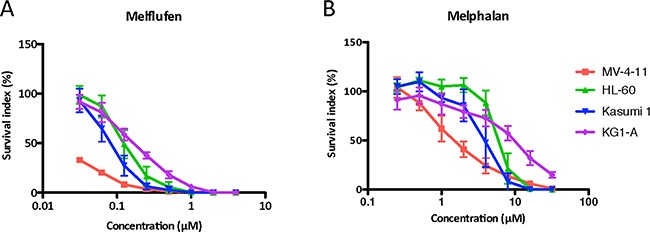
The cytotoxic activity (as concentration vs survival measured by FMCA) of melflufen **A**. and melphalan **B**. in AML cell lines MV-4-11, HL60, Kasumi and KGI-A. Mean values with SD of three independent experiments.

In the AML cell lines melflufen showed potent cytotoxic activity (Figure 1A) with IC_50_-values ranging from 0.018 to 0.17 μM (Table 1). Melphalan was considerably less potent (Figure 1B) and the highest ratio of IC_50_-values (melphalan/melflufen) was observed for the FLT-3 mutated MV-4-11 cell line.

**Table 1:** Mean IC_50_ values (with 95% confidence intervals) of melflufen and melphalan in 4 different AML cell lines (MV-4-11, HL60, Kasumi and KGI-A), AML patient samples and PBMCs from healthy donors

	melflufen IC_50_ (μM)	melphalan IC_50_ (μM)	IC_50_ ratio
MV4-11	0.018 (0.016-0.019)	1.7 (1.4-2.0)	94
HL-60	0.13 (0.11-0.14)	6.1 (5.5-6.9)	47
Kasumi	0.082 (0.074-0.091)	3.8 (3.3-4.5)	46
KG1-A	0.17 (0.15-0.18)	8.6 (7.1-10)	51
Patient samples	0.067 (0.051-0.088)	5.5 (4.7-6.5)	82
PBMC	0.5 (0.30-0.85)	9.7 (5.0-19)	19

The ratio (melphalan/melflufen) describes magnitude of superiority.

### Combinations in AML cell lines

**Figure 2:**
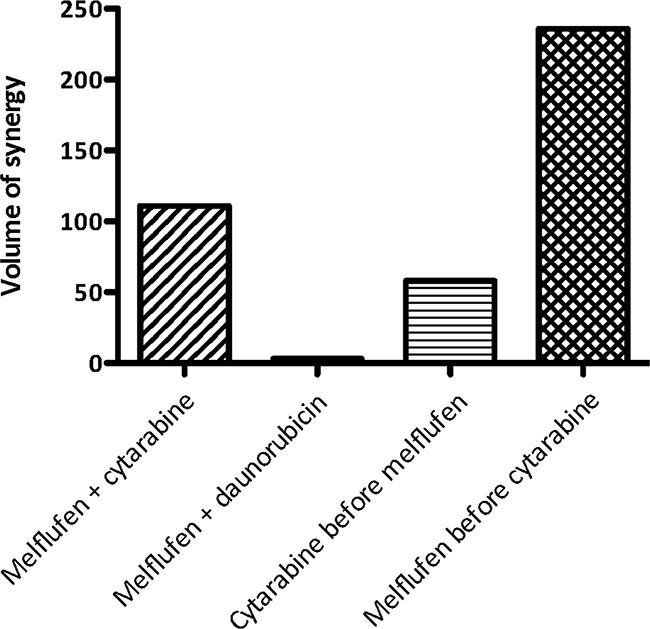
Combination effects of melflufen and cytarabine or daunorubicin in HL-60 cells The MacSynergy II program (Prichard) was also used to analyze the whole concentration matrix according to the Bliss independence model. The three-dimensional differential surface plot demonstrates synergy as peaks above a theoretical additive plane (positive volume of synergy), and antagonism as depressions below it (negative volume of synergy).

Volumes of synergy for melflufen combination effects with either cytarabine or daunorubicin on the inhibition of HL-60 cell line are shown in Figure 2. The combination between melflufen and cytarabine was shown to be highly synergistic when the drugs were added simultaneously, whereas only sub-additive interactions were observed for melflufen combinations with daunorubicin (Table 2). Volumes of synergy for melflufen+cytarabine was 111 and for melflufen+daunorubicin 3.1. When sequence dependence was tested for the melflufen-cytarabine combination, administrating melflufen 24 hours before cytarabine produced significantly stronger volume of synergy than the opposite sequence (236 vs 58, Figure 2).

**Table 2:** Combination activity of melflufen and cytarabine or daunorubicin in HL-60 for selected concentrations (calculated according to the multiplicative Bliss model)

Concentrationmelflufen(μM)	Concentrationcytarabine(μM)	Concentrationdaunorubicin(μM)	PI melflufen(%)	PIcytarabine(%)	PIcombination(%)	PIcombinationpredicted	Interaction
0.030	0.25	NA	47	30	71	63	S
0.030	0.50	NA	47	45	83	71	S
0.063	0.25	NA	71	30	83	79	S
0.063	0.50	NA	71	45	88	84	S
0.030	NA	0.013	47	40	54	68	SA
0.030	NA	0.025	47	58	71	77	SA
0.063	NA	0.013	71	40	74	83	SA
0.063	NA	0.025	71	58	78	88	SA

Abbreviations: PI; percent inhibition, S; Synergy, SA; Subadditive interaction.

### Dependency on exposure time and cellular density

**Figure 3:**
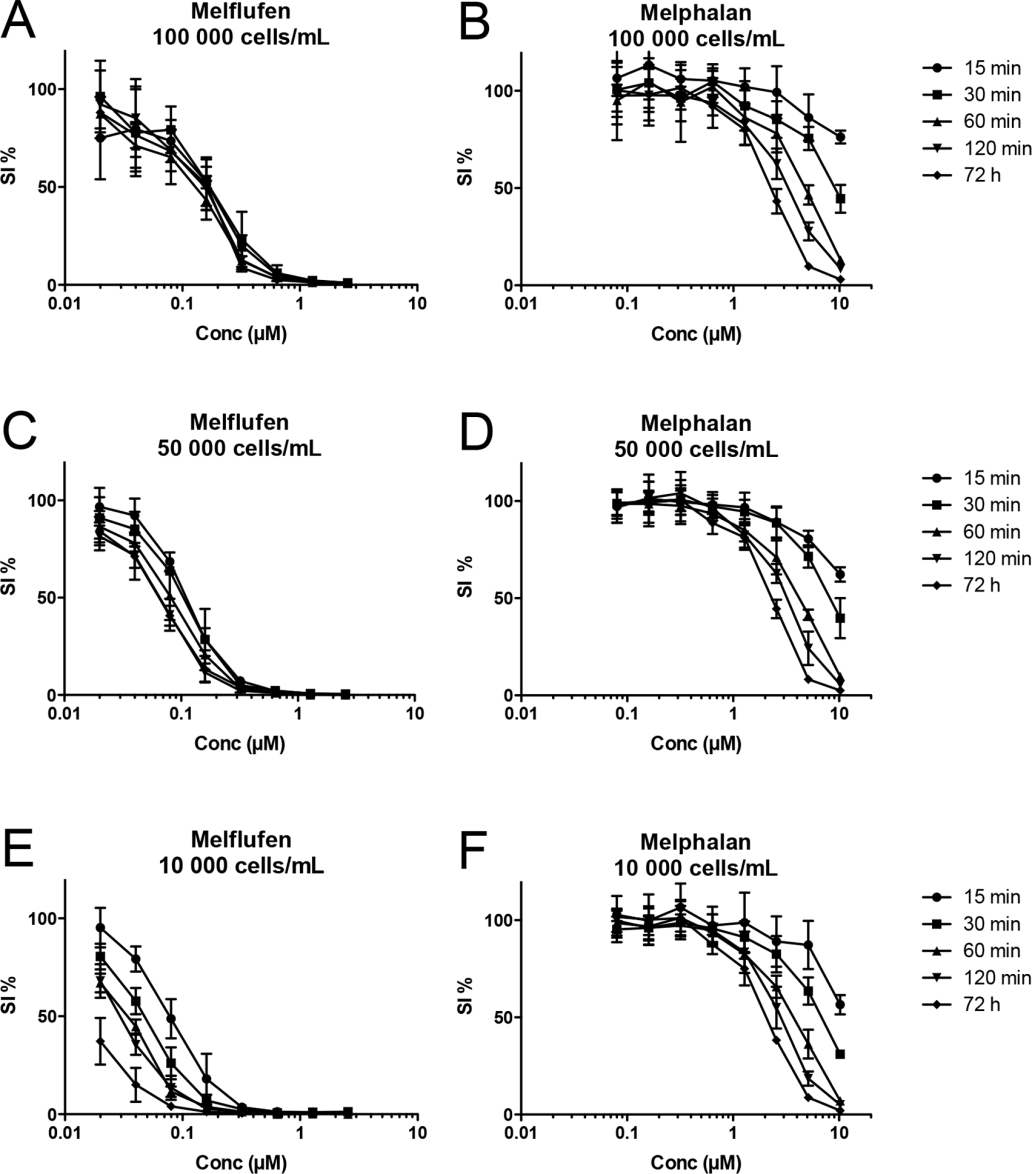
Time course for melflufen's accumulation and cytotoxic effect in CCRF-CEM leukemia cells The peptidase-potentiated effect results in a rapid accumulation of the active drug, and with decreasing cell densities **A-C-E**. there are more drug molecules per cell, and activity increases (curves shifts to the left). The rapid accumulation results in a competition for melflufen, and with high cell densities (A) maximum effect is obtained already after 15 minutes, i.e. cells have taken up all melflufen from the medium. When the cell number is lower (C and E) there is a time course for the accumulation, and longer exposure yields higher activity (i.e. lower IC50). For melphalan the situation is completely different as the absorption of the drug is slow; longer exposure time results in higher activity regardless if the cell density is high or low, suggesting rather slow absorption and a concentration equilibrium **B-D-F**.

Different exposure times and cellular densities were employed to study the time course for melflufens´s accumulation and cytotoxic effects in leukemia cells. Results presented in Figure 3 demonstrate that melflufen and melphalan behave very differently, and while melphalan's activity appear almost insensitive to changes in cell density but highly dependent on longer exposure, it is clear that the effect of melflufen hits the cells very rapidly if supported in excess.

### Patient samples and PBMCs

**Figure 4:**
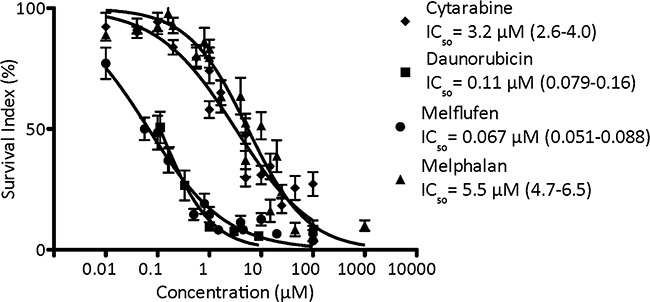
The cytotoxic activity (as concentration vs survival measured by FMCA) and mean IC50 (95% confidence intervals) of cytarabine, daunorubicin, melflufen and melphalan in AML patient samples Error bars denote SEM.

Primary cultures of patient tumor cells has not been used very often in the context of cancer drug screening and development, lthough *in vitro* assays performed on cells from different diagnoses may detect tumor-type specific activity. The mean cytotoxic activity of cytarabine (n= 78), daunorubicin (n= 15), melflufen (n= 82) and melphalan (n= 79) in 82 AML patient samples is shown in Figure 4. Melflufen was the most cytotoxic drug tested in patient cells with an average IC_50_ of 0.067 μM, followed by daunorubicin with an IC_50_-value of 0.11 μM, while cytarabine and melphalan had higher IC_50_ values of 3.2 μM and 5.5 μM respectively. The difference in cytotoxic activity of melflufen and melphalan in patient AML samples was high (82-fold, p<0.0001), in consistence with the results obtained from the cell lines.

There was a clear difference (p< 0.0001) of melflufen activity between patients with a low/intermediate/high-risk genetic profile for all three groups, and the activity of melflufen was higher (p< 0.0001) in patients succeeding 2-years survival (not shown). Although no significant differences in melflufen activity were obtained for the remaining stratification factors, there was a clear trend for higher activity of melflufen in chemo-naïve patients, in patients responding to subsequent therapy, debut and primary AML. The activity (expressed as logIC_50_) of melflufen vs melphalan weakly but significantly correlated (R^2^= 0.14, p= 0.0029), as expected for alkylating agents. In addition there was a slight but significant correlation vs doxorubicin (R^2^= 0.097, p= 0.015), consistent with its ability to induce DNA damage, but not vs amsacrine (R^2^= 0.00076, p= 0.85), cytarabine (R^2^= 0.0053, p= 0.57), etoposide (R^2^= 0.027, p= 0.21), imatinib (R^2^= 0.047, p= 0.076) or vincristine (R^2^= 0.016, p= 0.35) (Supplementary Figure 2).

When the activity of melflufen in primary cultures of AML cells obtained from patients was compared with normal PBMCs (n= 10; Table 1 ), a 7.5-fold higher (i.e. lower IC_50_) degree of cytotoxicity was observed in the AML cultures. The corresponding difference in sensitivity to melphalan was only 1.8-fold, indicating a more favorable therapeutic index for melflufen.

### Myeloid progenitor cells

When melflufen was tested on immature CD34^+^ progenitor cells (FMCA-GM7) and a more differentiated CD34^+^ derived cell population (FMCA-GM14) a very similar sensitivity was observed with IC_50_-values of 0.028 and 0.029 μM, respectively, in contrast the stem cell like GM7 cells were less sensitive to melphalan compared to GM14 cells with IC_50_-values of 1.9 and 0.68 μM, respectively. The IC_50_ ratio between melflufen and melphalan was 68 and 23 for GM7 and GM14 cells, respectively.

### Xenografts of patients derived tumor samples

**Figure 5:**
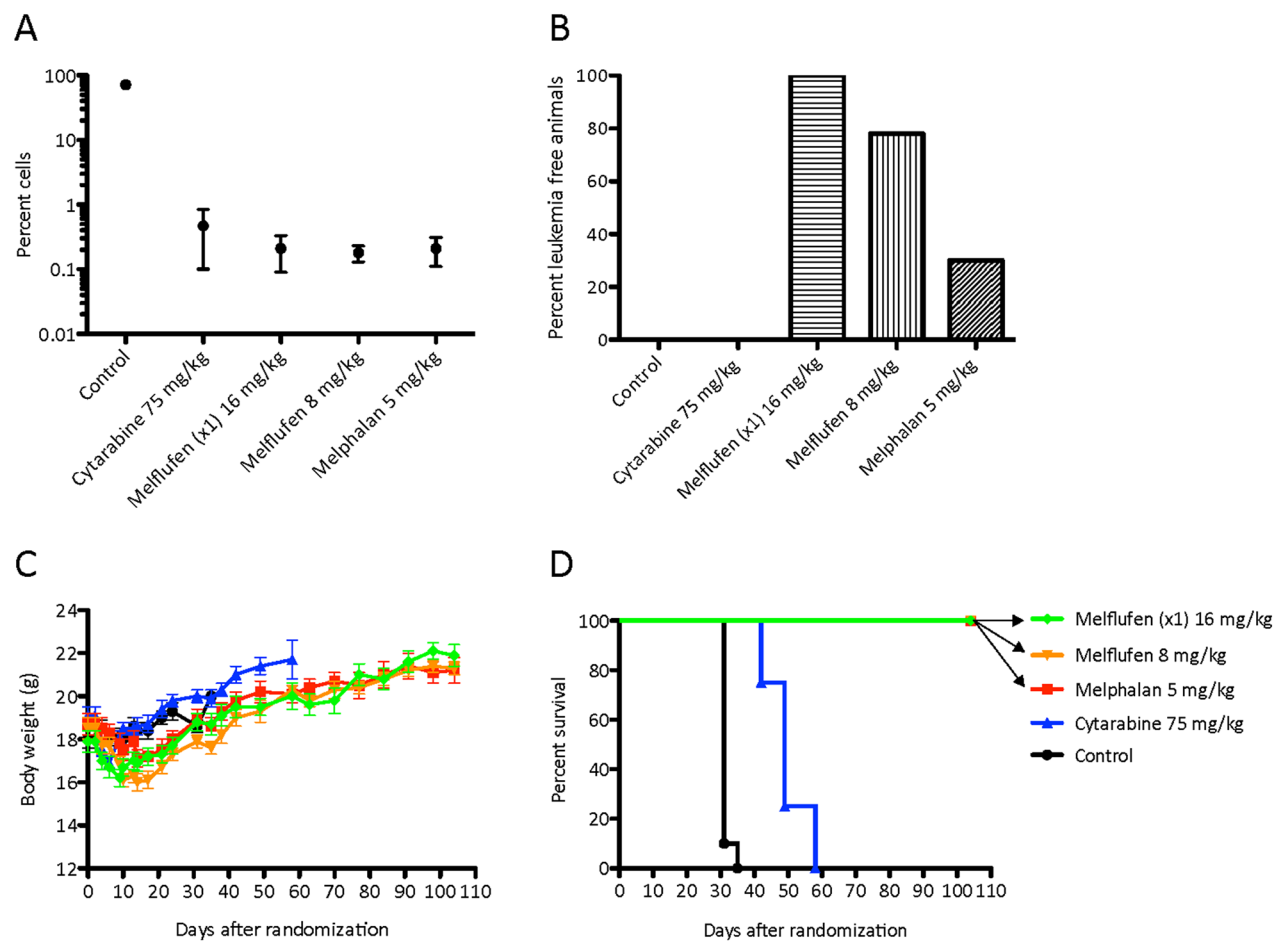
*In vivo* effects of cytarabine and melflufen Effect presented as percent of HLAABC positive AML-ps cells in blood (determined by FACS) on day 30 **A**., proportion of animals free of leukemia at end of experiment on day 104 **B**., change in mean body weight of remaining live animals **C**. and median Kaplan-Meier survival curve **D**. Error bars denote SD.

The *in vivo* study with AML-ps xenograft showed significant effects of melflufen regarding both leukemia control and survival. The amounts of circulating HLA-ABC (eg leukemic) cells at day 30 after inoculation were significantly (p<0.001) reduced vs control for all drugs, the number of positive cells in the blood from melflufen and melphalan treated mice was slightly lower (non-significant) than from cytarabine treated mice (Figure 5a).

Upon macroscopic evaluation, at euthanization on day 104, all mice treated with melflufen (16 mg/kg single dose) were considered tumor free, with normal size organs, and in the repeated melflufen group (8 mg/kg x 4), 7/9 mice were considered tumor free, while two had splenic abnormalities (Figure 5b). Only 3/10 mice treated with melphalan could be considered tumor free (splenic abnormalities in 7).

All animals in the treatment groups lost weight at some stage after treatment. The differences were not significant, and the mean maximal weight loss was 11.4% (day 6) in the cytarabine group, and 9.5% (day 9), 13.5 % (day 14), and 8.6% (day 14) in the melflufen single, repeated and melphalan group, respectively (Figure 5c).

The median survival time (MST) of vehicle-treated control animals was 34 days, and in the cytarabine (75 mg/kgx1 for five days during 2 weeks) group 49 days. In contrast, all melflufen (16 mg/kg single dose and 8 mg/kg) and melphalan (5 mg/kg) treated animals were still alive at day 104 when the study was terminated as per protocol (i.e. at 3x control MST; Figure 5d).

## DISCUSSION

The novel alkylating agent melflufen possess significantly higher *in vitro* and *in vivo* anticancer activity compared to melphalan despite similar alkylating capacity, i.e. one bis-(chloroethyl)-amino unit per molecule. *In vitro* studies have shown that melflufen is rapidly taken up into the cytosol of tumor cells followed by intracellular hydrolysis resulting in the subsequent release of alkylating moieties (e.g. melphalan), reaching considerably higher concentrations compared to direct treatment with melphalan [5]. This potentiation is mediated by the action of aminopeptidases, including APN/CD13, which is extensively expressed on leukemic blasts in all AML subtypes [6, 10], but also on normal progenitor and stem cells [21] and therefore hematotoxicity of APN-targeted drugs could be expected. It was recently shown that monoclonal antibodies against APN could induce apoptosis in AML cells independently of APN enzymatic activity [11], which underlined APN as a possible drug target in AML [8]. Preclinical safety studies of melflufen in rats and mice show a toxicity profile common to alkylating agents with hematotological toxicity being dose-limiting. The estimated maximum tolerated dose (MTD) of melflufen in rodents was close to that previously reported for melphalan (Data on file, Oncopeptides AB Stockholm, Sweden, and [16]). Melflufen is presently evaluated in phase II clinical trial for treatment of multiple myeloma. Promising clinical results was recently reported with 67 % of patients showing objective responses (11 partial and 3 minor responses) in 21 patients (refractory to iMIds and proteasome inhibitors) evaluable for efficacy. Notably, clinically beneficial partial responses was observed also in patients refractory to high dose melphalan [22]. Given the strong indication of clinical activity in the ongoing phase II study, it seems of outmost importance to identify other potential target diagnoses for the clinical development of the compound. In the present study we demonstrate significant activity of melflufen in AML both in cell line models and primary cultures of AML cells obtained from patients as well as in PDX model of AML *in vivo*.

In the AML cell line panel the most sensitive was FLT3 mutated MV-4-11 cells. The receptor tyrosine kinase FLT3 is predominantly expressed on pluripotent hematopoietic cells and has a role in the regulation of proliferation and differentiation [23, 24]. In AML FLT3 is commonly expressed on leukemic blasts. Activating mutations of FLT3, including FLT3 internal tandem duplications (FLT-ITD) are among the most common genetic alterations in AML (approximately 30% of the patients), and FLT3-ITD positivity has been associated with increased relapse rate and diminished overall survival [25, 26]. Although MV-4-11 is often considered as a chemosensitive cell line, it is encouraging to note that melflufen is very active against cells displaying this mutation. In this respect, it is also encouraging to note the high activity of melflufen in primary cultures of AML cells from patients, 80-fold more potent than melphalan. In addition, melflufen was six-fold more potent in the primary AML cultures compared to normal PBMC cultures, indicating a more beneficial *in vitro* therapeutic index. Melphalan on the other hand was only two-fold more potent in AML compared to PBMCs. It should be noted that both PBMCs and AML samples proliferate poorly under the current assay conditions. The high therapeutic index (AML vs PBMC) observed for melflufen could potentially be related to APN expression and activity in AML cells. It has been convincingly shown that melflufen is a substrate for APN, yielding intracellular melphalan as the hydrolysis product. It is therefore tempting to speculate that the enzymatic reaction is preferentially occurring in or at the proximity of APN overexpressing AML blasts, thus leading to potentiated melflufen activity in these cells. It is also worth noting that the IC_50_ obtained in AML cells is well below clinically achievable plasma concentrations, i.e. C_max_> 1 μM [27].

In patients with hematologic malignancies, allogeneic hematopoietic stem cell transplantation (HSCT) using reduced-intensity conditioning with regimens including melphalan has been evaluated in clinical studies [28–30]. In proliferating CD34^+^ progenitor cells, the melflufen IC_50_ was comparable to that found in the low proliferating primary AML cell cultures. The IC_50_ ratios between melflufen and melphalan for stem cell like GM7 and differentiated GM14 cells were lower than that observed in primary AML cells. It was interesting to note that melflufen was equally active in the two normal cell models whereas the stem cell like GM7 was less sensitive to melphalan. The limited long-term efficacy of many current AML therapies (i.e. high frequency of relapse) has often been attributed to the inability of standard chemotherapeutic agents to target AML stem/progenitor cells. This activity of melflufen on the immature (stem cell like) model might contribute to the superior clinical activity over melphalan and could indicate a potential role as a replacement of melphalan in stem cell transplantation [28–30]. Interestingly, the side effect profile of intravenous melflufen, including its myelotoxicity, observed in a clinical phase I study, appeared similar to that of melphalan treatment, both qualitatively and quantitatively [27].

In the present study, melflufen was also tested in combination with cytarabine and daunorubicin, two cornerstone drugs in the treatment of de novo AML. Synergy was observed for the melflufen-cytarabine combination, whereas only subadditive interactions were found for the melflufen-daunorubicin counterpart, when investigated in AML cell lines. The observed synergy with cytarabine is encouraging since this compound is often used also after relapse, a therapeutic situation where melflufen is likely to be evaluated. Interestingly the synergistic interaction with cytarabine appeared to be sequence dependent with increased synergy when melflufen was added before cytarabine. A plausible explanation is that melflufen may increase the proportion of cells in S-phase during the first 24 (-48) hours after exposure, as recently shown in lymphoma cell lines [31], which would increase cytarabine potency. Conversely, cell cycle arrest induced by cytarabine may affect the activity of other drugs used in combination [32], but as an alkylator melflufen is not likely cell cycle specific in its cytotoxicity. Results suggest that melflufen may be further investigated in relapsed patients using a timed sequential combination. In fact several prior examples show that such an approach may be reasonable for development of new AML therapies [33–35].

Finally melflufen induced significant activity in a patient derived xenograft model of AML, and animals treated with melflufen survived more than 3 times longer than control animals, and when terminated at the end of the experiment a majority of the melflufen treated animals were without signs of residual leukemia (at macroscopical examination). PDX models has been shown to reflect the clinical situation with improved predictions of clinical utility [36] which in combination with the *in vitro* results makes melflufen a strong candidate for clinical evaluation.

In conclusion, melflufen demonstrates high and significant preclinical activity in AML and further clinical evaluation seem warranted in this disease

## MATERIALS AND METHODS

### Drugs

Melflufen (kind gift from Oncopeptides AB, Stockholm, Sweden) was dissolved in dimetylsulfoxid (DMSO) and further diluted in sterile water or phosphate buffered saline (PBS; Sigma-Aldrich, Saint Louis, Missouri, USA). For the patient derived xenograft (PDX) model melphalan was obtained as Alkeran^®^ were obtained from the State-owned Pharmacy Chain (Apoteket AB, Uppsala, Sweden), and for fluorometric microculture cytotoxicity assay (FMCA) it was bought as a pure chemical from Sigma Aldrich or as Alkeran^®^. All dilutions with water/PBS were made immediately prior to the start of the experiments to minimize the influence of compound hydrolysis. The standard drugs amsacrin, cytarabine (ara-c) daunorubicin, doxorubicin, etoposide, vincristine (Sigma Aldrich) and imatinib (LC Laboratories, Woburn, Massachusetts, USA) were dissolved in DMSO and diluted in PBS or sterile water immediately prior to start of the experiments.

### Cell lines

AML cell lines MV-4-11 (FLT3-ITD mutated), HL60 (FLT3 wild type), Kasumi (translocation 8:21), and KGI-A were obtained from American Type Culture Collection (ATCC; Wesel, Germany) and were cultured in Dulbecco´s Modified Eagles medium (DMEM) supplemented with 10% heat-inactivated fetal bovine serum (HI-FCS), 100 U/mL penicillin, 100 μg/mL streptomycin and 2 mmol/L L-glutamine (2% pest/glut; Sigma-Aldrich). The cell lines were grown in 75 cm^2^ culture flasks (TPP, Trasadingen, Switzerland) at 37°C in a humidified atmosphere of 95% air and 5% carbon dioxide. The cells were counted in a Bürker chamber and diluted in cell culture medium to a concentration of 50 000 cell/mL for use in the fluorometric microculture cytotoxicity assay (FMCA).

### Patient samples

Leukemic cells ware obtained from 82 bone marrow or peripheral samples in 73 patients (median age 54 yrs) with AML. The sampling was approved by the Ethics committee of Uppsala University. As previously described by Blom *et al*. [37], AML cells and peripheral blood mononuclear cells (PBMC) from healthy donors were isolated from bone marrow or peripheral blood by 1.007 g/ml histopaque (Pharmacia Biotech, Uppsala) density gradient centrifugation. Cells were counted using toluidine blue and resuspended in RPMI 1640 cell culture medium, supplemented with 10% HI-FCS and 2% pest/glut (all Sigma-Aldrich), to a concentration of 0.4 × 10^6^ cells/mL for use in the FMCA. A proportion of ≥70% viable blast cells were set as a prerequisite. Melflufen was tested on all of the 82 samples, 79 of these was tested against melphalan, 78 against cytarabine and 15 against daunorubicin. Patient background information could be extracted from the medical records for a majority of the samples (67 of 82). The material was subsequently grouped for the factors: prior chemotherapy (given (n= 40)/not given (n= 19)) response to subsequent chemotherapy (complete response (n= 22)/partial response (n= 11)/progressive disease (n= 46)); patient survival after sampling (<2 years (n= 50)/>2 year (n= 15)); genetic risk-profile according to the ELN-criteria [38] (high (n= 20)/intermediate (n= 25)/low (n= 9)); disease status (debut (n= 38)/relapse (n= 25)) and origin (de novo (n= 52)/secondary AML (n= 6)). One-way ANOVA (for three variables) or unpaired t-test (for two variables) was used in Graph Pad Prism 5 Software Package (Graph Pad, San Diego, California, USA) to determine differences in mean logIC_50_.

### Myeloid CD34^+^ umbilical cord blood cells

These assays have been described in detail previously [39]. Cryopreserved human umbilical cord blood CD34^+^ progenitor cells were purchased from 3H Biomedical AB (Cat no. 3H-902-10; Uppsala, Sweden). After isolation, the CD34^+^ progenitor cells (92–95% purity) were cryopreserved in −150 **°**C in fetal bovine serum and DMSO. The cells were rapidly thawed in a 37 **°**C water bath and suspended in a cell thawing media (3H Biomedical), centrifuged at 1000 rpm for 10 min and cultured in cytokine supplemented stem cell culture medium for granulocytopoietic lineage differentiation, including stem cell factor (SCF), interleukin-3 (IL-3), Flt ligand (FL), granulocyte colony-stimulating factor (G-CSF) and granulocyte-macrophage colony-stimulating factor (GM-CSF) (Cat. no. 900-30-50, 3HBiomedical), at 37 **°**C, 5% CO_2_. Two types of assay setups were performed to determine the drug sensitivity of the cells, with drug treatment starting at different degrees of cell differentiation. The cells were stimulated towards the granulocyte-macrophage lineage in a 7-day assay (FMCA-GM7), where cells were exposed to drugs day 0–7 of differentiation and cell survival was analyzed with the FMCA on day 7 [39]. In the 14-day assay (FMCA-GM14), the cells were first cultured with stimulating cytokines in a U-shaped 96-well microplate in absence of drugs. After 7 days of culturing the committed cells were resuspended, plated in a 384-well microplate and exposed to drugs during day 7–14 of differentiation with cell survival analyzed on day 14 [39].

### Fluorometric microculture cytotoxicity assay

The cytotoxic efficacy of melflufen, melphalan, cytarabine and daunorubicin was analyzed by the total cell kill assay fluorometric microculture cytotoxicity assay (FMCA). The method is based on the fluorescence generated from the hydrolysis of fluoresceindiacetate (FDA) to fluorescein by cells with intact cell membranes, previously described by Larsson *et al*. [40] and as a protocol by Lindhagen *et al*. [41]. In short, 45 μL cell suspension per well were seeded into drug containing (5 μL/well) 384-well Nunc microtiter plates (Thermo Scientific, Roskilde, Denmark). Or, 50 μL cell suspension per well were seeded into a 384-well microtiter plates (Nunc) and incubated for 24 hours (37°C, humidified atmosphere, 5% CO_2_), after which 2.5 nl portions/well of drug stock in anhydrous DMSO were added to the cells using Echo 550 Liquid Handler (Labcyte, Dublin, Ireland). The plates were thereafter incubated, without medium change, in a humidified atmosphere, at 37°C, containing 95% air and 5% CO_2_ for 72 hours. Cells were washed once with PBS, fluorescein diacetate (FDA; Sigma-Aldrich) were added (50 μL, 10 μg/mL) and plates were incubated for 60 minutes before measurement of fluorescence (485/538 nm for excitation and emission respectively) in a FLUOstar Omega (BMG Labtech, Offenburg, Germany). The measured fluorescence is directly proportional to the amount of viable cells with intact cell membrane. Survival index (SI%) for each drug concentration was calculated, expressed as percentage of test wells divided by control cultures, with blank values subtracted. For cell line assays, experiments were performed three times. From the mean SI%-logConcentration curves the half maximal inhibitory concentration (IC_50_) was determined using non-linear regression analysis in GraphPad Prism. To study the time course for melflufen and melphalan´s accumulation in CCRF-CEM leukemia cells a set of experiments with varying cell number (10-100 000 cell / mL) were performed in 96-well microtiter plates with limited exposure times (i.e. adding a wash) before 72 hr of total exposure.

### Combinations

Melflufen in combination with standard AML-drugs cytarabine and daunorubicin was investigated in the HL-60 (acute promyelocytic leukemia) cell line. The cytotoxicity of the compounds were tested with the FMCA method alone and in combinations in a 7×9 concentration matrix using 384-well plates with 4 replicates per sample, in two independent experiments. The concentrations ranges were 0.0156-1, 0.004-1 and 0.0004-0.1 μM for melflufen, cytarabine and daunorubicin, respectively. The effect of the combinations was evaluated for concentrations showing a percent inhibition (PI) between 30-76% with the multiplicative method of Bliss which allows measurement of antagonistic, additive, subadditive and synergistic interactions of drug combinations [42]. The MacSynergy II program (Prichard) was also used to analyze the whole 7×9 matrix according to the Bliss independence model [42], where the effect of the combination is determined by subtracting the experimental values from the theoretical additive values for every combination in the matrix [43]. A three-dimensional differential surface plot demonstrates synergy as peaks above a theoretical additive plane and antagonism as depressions below it [43]. Volume above the calculated additive plane (synergy) and log volume was calculated. As suggested by Prichard et al. [44], such data sets should be interpreted as follows: volumes of synergy or antagonism at values of <25 are insignificant, 25–50-minor but significant, 50–100-moderate and probably important *in vivo*, and >100-strong and likely to be important *in vivo*.

### Xenografts of patients derived tumor samples

The disseminated patient derived xenograft (PDX) study was performed at Accelera Srl, (Nerviano, Milan, Italy) according to a standardized protocol. Animal facility and in-vivo procedures had the highest ethical standards accredited by Association for Assessement and Accreditation of Laboratory Animal Care (AAALAC) International. The environment and quality standards were in compliance with International OECD principles on GLP, although not audited by the internal Quality Assurance staff. The antitumor activity of melflufen was investigated in AML primary sample cells (AML-ps; CD34^+^, CD33^+^, HLA-ABC*) transplanted female severe combined immunodeficiency (SCID) mice. The AML-ps cells was obtained in SCID mice by injecting bone marrow blasts from a 61 yo male patient in his third leukemic relapse (FAB M1), CD13+, CD33+, CD34+, CD45+, HLA ABC+, 46XY/49 XY, -8 -9 +11 +12 +21 +21 +21. The maintenance of these characteristics was routinely checked at the CRO facility.

Briefly, 5×10^6^ AML-ps cells were injected i.v. in SCID mice. After 2 days (preventive protocol) mice were randomized in different experimental groups (10-12 animals per group) and treatment started. Vehicle, melflufen and melphalan were administrated intravenously. A 5% glucose solution was used as control and administered twice weekly (2QW x 2W). Melflufen was dissolved in NN-dimethylamide (DMA) to a concentration of 50 mg/mL, and within 30 minutes prior to administration further diluted with 5% glucose solution. Melflufen was either administrated as single injection (16 mg/kg x 1), or twice a week for two weeks (8 mg/kg, 2QW x 2W), doses were selected from previous animal studies and were known to be tolerable in mice. On a molar level 5 mg/kg of melphalan equals 8 mg/kg of melflufen. Alkeran^®^ (melphalan) was dissolved with the accompanied solvent and further diluted with 5% glucose solution within 30 minutes prior to administration, schedule matched the melflufen repeated (5 mg/kg, 2QW x 2W). Doses of melphalan (5 mg/kg x 4) and melflufen (8 mg/kg x 4) were selected to be ‘equimolar’, i.e. to deliver the same amounts of alkylating *bis-(chloroethyl)amino* units to both groups for direct comparison of activity. The insertion needle was rinsed with saline or glucose prior to retraction to avoid local inflammatory reactions. Cytarabine was used as a positive control, and administered intraperitoneally (according to company standard model) daily on weekdays (75 mg/kg, QDx5 × 2W).

Mice mortality, clinical signs and food consumption were monitored daily, body weight was controlled every 3 days. Cytotoxic efficacy was evaluated in terms of survival compared to control (glucose solution). Toxicity was evaluated on the basis of the body weight reduction and all organs were macroscopically examined upon sacrifice. Leukemic mortality was the main endpoint, in addition the total amount of circulating leukemia cells was determined by flow cytometry (Fluorescence Activated Cell Sorter; FACS) after inoculation of Human Leukocyte Antigen (HLA)-ABC on day 30, and morphological signs of leukemia examined at autopsy. During the assay development it demonstrated that leukemic infiltrates were observed in the bone marrow and liver from day 15, and in spleen, stomach, lung and kidneys at day 29.

## SUPPLEMENTARY MATERIALS FIGURES


