# Supplementary material for: In vitro and in vivo anti-leukemic activity of the peptidase-potentiated alkylator melflufen in acute myeloid leukemia
Source: Oncotarget. 2016 Dec 10;8(4):6341–52. doi: 10.18632/oncotarget.13856 (PMC5351636; doi:10.18632/oncotarget.13856)
Supplement: Supplementary file 1 [file oncotarget-08-6341-s001.pdf]

In vitro and *in vivo* anti-leukemic activity of the peptidase-potentiated alkylator melflufen in acute myeloid leukemia

SUPPLEMENTARY FIGURES

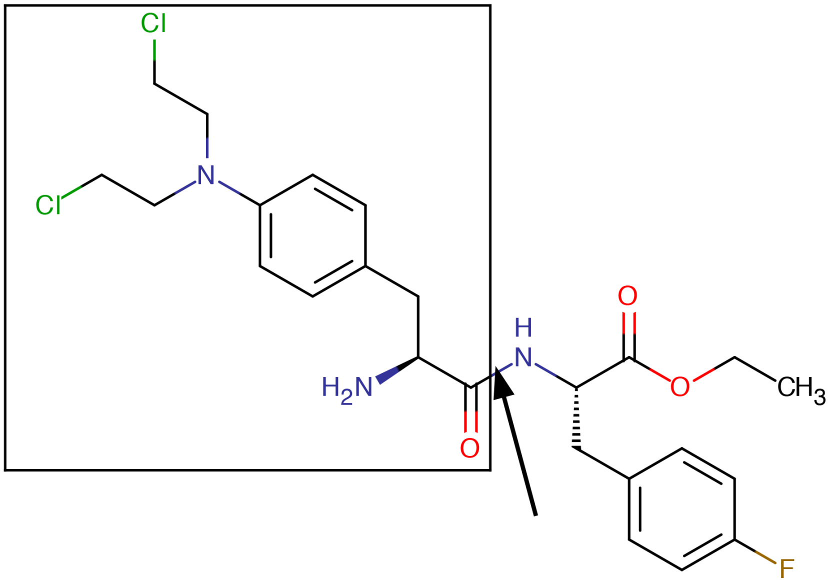

Supplementary Figure S1: Chemical structure of melflufen. Arrow show peptide bond susceptible to hydrolysis by e.g. APN. Structure element in square corresponds to melphalan.

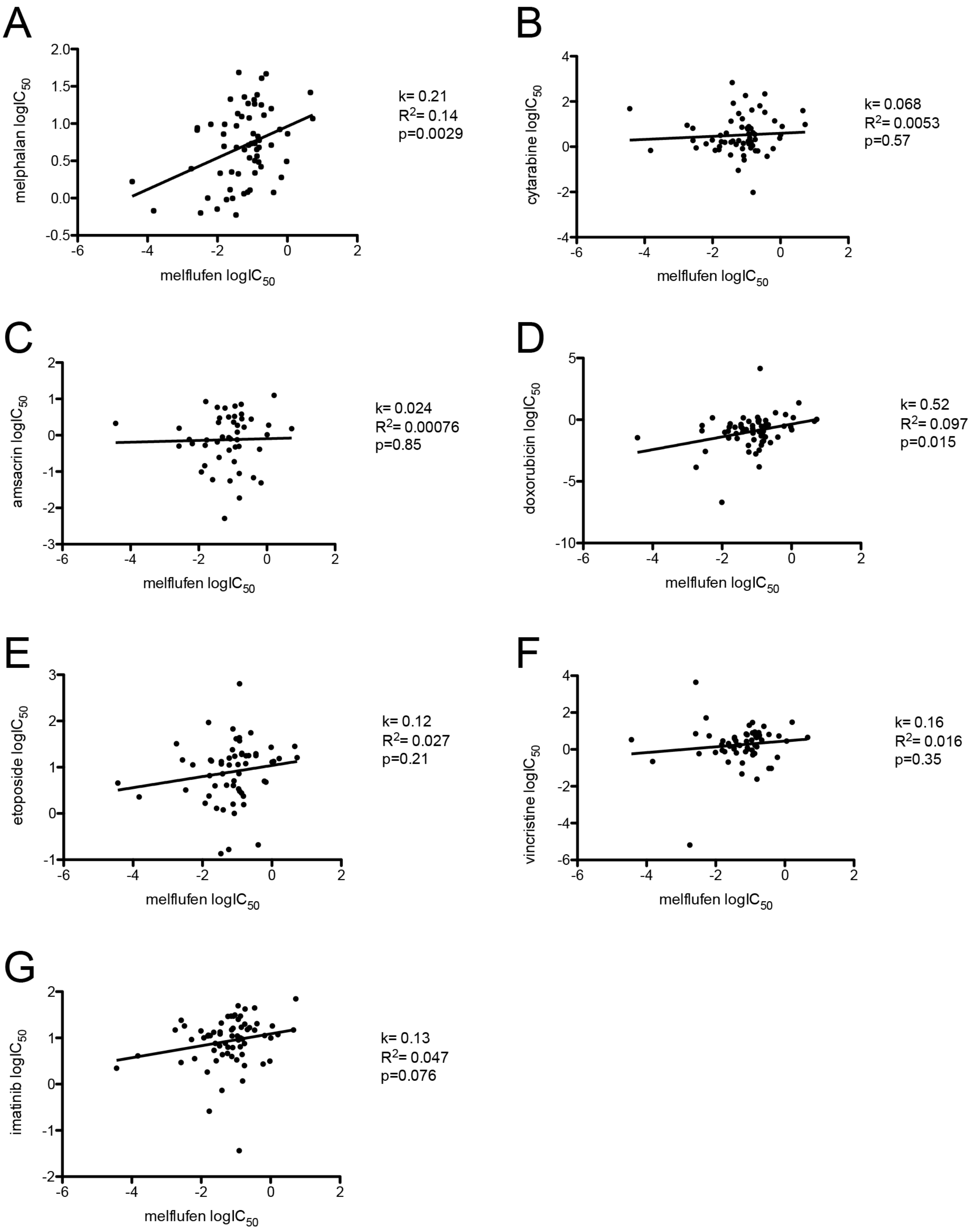

Supplementary Figure S2: The correlation of melflufen vs. A. melphalan (R^2= 0.14), **B.** cytarabine (R^2= 0.0053), **C.** amsacrine (R^2= 0.00076), **D.** doxorubicin (R^2= 0.097), **E.** etoposide (R^2= 0.027), **F.** vincristine (R^2= 0.016) and **G.** imatinib (R^2= 0.047) in primary cultures of AML cells derived from patients.
